# Annual acknowledgement of manuscript reviewers

**DOI:** 10.1186/2049-9957-3-4

**Published:** 2014-02-11

**Authors:** Xiao-Nong Zhou

**Affiliations:** 1 National Institute of psitic Diseases, Shanghai, China CDC

## Abstract

**Contributing reviewers:**

The editors of *Infectious Diseases of Poverty* would like to thank all our reviewers who have contributed to the journal in Volume 2 (2013).

## 

Isabella Aboderin

Kenya

Rabiul Ahasan

Malaysia

Paul Akpa

Nigeria

Pascale Allotey

Malaysia

Hesham Al-Mekhlafi

Malaysia

Gloria Ansa

Ghana

Tania C de Araujo-Jorge

Brazil

Solome Kiribakka Bakeera

Uganda

Yan Bi

Australia

Franziska Bieri

Australia

Gautam Biswas

Switzerland

Diana Boraschi

Italy

Sanaa Botros

Eygpt

Christine Budke

United States of America

Nora Cardona-Castro

Colombia

Edith Certain

France

Kwang Poo Chang

United States of America

Himanshu Chaturvedi

India

Zhao Chen

China

Fidelis Cho-Ngwa

Cameroon

Archie Clements

Australia

Ian Gillespie Cook

United Kingdom

Rodrigo Correa-Oliveira

Brazil

Carlos Costa

Brazil

Iliano Coutinho-Abreu

United States of America

Filippo Curtale

Uganda

Fernando De Maio

United States of America

Uwem Ekpo

Nigeria

Tambo Ernest

Benin

Ana Faleiros

Brazil

Chia-Kwung Fan

Taiwan

Luis Fariña

Spain

Basu G

India

Carlos Graeff-Teixeira

Brazil

Sian Griffiths

Hong Kong

Yoshiaki Gu

Japan

Wei Guo

China

Maria G Guzman

Cuba

Margaret Gyapong

Ghana

Xiumin Han

China

David Heath

New Zealand

Fang Huang

China

Yixin Huang

China

James Hughes

United States of America

Qing-Wu Jiang

China

Nicholas Johnson

United Kingdom

Malcolm Jones

Australia

Masanori Kai

Japan

Faiz Kermani

France

Eili Klein

United States of America

Merav Kliner

United Kingdom

Randall Kramer

United States of America

Agnes Kurniawan

Indonesia

Eliningaya Kweka

Tanzania

Tiaoying Li

China

Song Liang

United States of America

You-Sheng Liang

China

Lifu Liao

China

Jiming Liu

Hong Kong

Da-Bing Lu

China

Hongzhou Lu

China

Stephen Luby

United States of America

Zhao-Rong Lun

China

Sara Lustigman

USA

Shan Lv

Switzerland

Ye Ma

China

Pascal Magnussen

Denmark

Zuohua Mao

China

Tanguy Marcotty

Belgium

Don McManus

Australia

John Miller

Zambia

Michele Miller

United States of America

David Molyneux

United Kingdom

Susana Nery

Timor-Leste

Dinesh Neupane

Denmark

Jintana Ngamvithayapong-Yanai

Thailand

Nobuyuki Nishikiori

Philippines

Solomon Nwaka

Switzerland

Chinekwu Obidoa

United States of America

Ayo MJ. Oduola

Nigeria

David Ofori-Adjei

Ghana

Chinelo Okwuanaso

Nigeria

Ole Olesen

Belgium

Eric Ottesen

United States of America

Hong-Juan Peng

China

Xiaopeng Qi

China

Men-Bao Qian

China

Zhongdong Qiao

China

Daniel Reidpath

Malaysia

Gustavo Romero

Brazil

Bishnupada Roy

India

Moussa Sacko

Mali

Anna Seale

United Kingdom

Babar Tasneem Shaikh

Pakistan

Vinod Sharma

India

Benyun Shi

China

Yali Si

Netherlands

Marzieh-baygom Siahpoosh

Iran

Dhirendra Singh

United States of America

Johannes Sommerfeld

Switzerland

Banchob Sripa

Thailand

Peter Steinmann

Switzerland

Ernest Tambo

South Africa

Qing Tang

China

Sheng-lan Tang

USA

Marcel Tanner

Switzerland

Metoh Theresia

China

Jeanette Vega

USA

Elvina Viennet

Australia

Shanqing Wang

China

Yong Wang

China

Mohammad Wazir

Pakistan

Yi Wen

China

Zhong-Dao Wu

China

Blair J. Wylie

United States of America

Shang Xia

China

Lihua Xiao

United States of America

Jing Xu

China

Lili Xu

China

Guojing Yang

China

Peiling Yap

Switzerland

Sun Yi

China

Nasser Yousif

United States of America

Sen-Hai Yu

China

Liping Yuan

China

Yongyuth Yuthavong

Thailand

Yohannes Zenebe

Ethiopia

Haobing Zhang

China

Zaixing Zhang

Solomon Islands

Jinkou Zhao

Switzerland

Shuisen Zhou

China

Xia Zhou

China

Xiao-Nong Zhou

China

Yibiao Zhou

China

Huaimin Zhu

China

Jakob Zinsstag

Switzerland

Guanyang Zou

China

Dejan Zurovac

Kenya

